# Implications of therapy interruption on monthly migraine days and modified migraine disability assessment in patients treated with erenumab for chronic and episodic migraine: SQUARE study interim results

**DOI:** 10.1007/s00415-024-12470-6

**Published:** 2024-06-13

**Authors:** Andreas R. Gantenbein, Christophe Bonvin, Christian P. Kamm, Christoph J. Schankin, Chiara Zecca, Dominik Zieglgänsberger, Gabriele Susanne Merki-Feld, Heiko Pohl, Nicole Rudolph, Philippe Ryvlin, Reto Agosti, Elisabeth Schäfer, Ina Meyer, Monika Kulartz-Schank, Michael E. Arzt

**Affiliations:** 1 Department of Neurology and Neurorehabilitation, ZURZACH Care, Quellenstrasse 34, CH-5330 Bad Zurzach, Switzerland; 2 Hôpital du Valais, Sion, Switzerland; 3 Department of Neurology, Inselspital, University Hospital Bern, University of Bern, Bern, Switzerland; 4 Neurocenter, Lucerne Cantonal Hospital, Lucerne, Switzerland; 5 Neurocenter of Southern Switzerland, Ente Ospedaliero Cantonale, Lugano, Switzerland; 6 Faculty of Biomedical Sciences, University of Southern Switzerland, Lugano, Switzerland; 7 Department of Neurology, Hospital St. Gallen, St. Gallen, Switzerland; 8 Department of Reproductive Endocrinology, University Hospital Zurich, Zurich, Switzerland; 9 Department of Neurology, University Hospital Zurich, Zurich, Switzerland; 10 Department of Neurology, Hospital Münsterlingen, Münsterlingen, Switzerland; 11 Department of Clinical Neurosciences, CHUV, Lausanne, Switzerland; 12 Kopfwehzentrum Hirslanden, Zurich, Switzerland; 13 Novartis Pharma Schweiz AG, Rotkreuz, Switzerland; 14 Present Address: Janssen-Cilag AG, Zug, Switzerland

**Keywords:** CGRP, Migraine, Break, Real world evidence, RWE, Erenumab

## Abstract

**Background:**

There are limited real-world data in Switzerland examining the impact of erenumab, a fully human IgG2 monoclonal antibody targeting the calcitonin gene-related peptide (CGRP) receptor, on migraine-related quality of life.

**Objective:**

This 18-month interim analysis of 172 patients with episodic or chronic migraine from the SQUARE study provides first prospective insights on the impact of mandatory erenumab treatment interruption, following Swiss-reimbursement requirements, in a real-world clinical setting in Switzerland.

**Findings:**

Recruited patients receiving 70 or 140 mg erenumab underwent treatment interruption on average 11.2 months after therapy onset with a mean duration of 4 months. There were sustained improvements in mean monthly migraine days (MMD) and migraine disability (mMIDAS) during initial treatment with erenumab. Treatment interruption was associated with a temporary worsening of condition. Symptoms ameliorated upon therapy reuptake reaching improvements similar to pre-break within 3 months.

**Conclusions:**

Treatment interruption was associated with a temporary worsening of condition, which improved again after therapy restart.

## Introduction

Erenumab, a fully human IgG_2_ monoclonal antibody targeting the calcitonin gene-related peptide (CGRP) receptor, was approved in Europe for the preventive treatment of migraine in adults in 2018 [1]. In the context of routine medical care in Switzerland, there are limited real-world data examining the impact of erenumab on migraine-related quality of life in episodic (EM) and chronic migraine (CM) patients in Switzerland.

To fill this gap, the non-interventional study SQUARE (Swiss QUality of life and healthcare impact Assessment in a Real-world Erenumab treated migraine population) investigates the effects of erenumab treatment on patient-reported quality of life and migraine-related impairment, as well as treatment satisfaction and persistence in a real-world environment. The first results published recently showed that from baseline to month 6, erenumab significantly improved the Headache Impact Test (HIT-6™) score and monthly migraine days (MMD), along with other migraine-related parameters and quality of life measures, such as the modified Migraine Disability Assessment (mMIDAS) among others, in patients with both episodic (EM) and chronic migraine (CM) [2].

Non-interventional studies often do not focus on country-specific regulations and their effect on treatment patterns are rarely considered. An interesting aspect of this study makes one of the unique requirements of Switzerland’s healthcare and insurance system, i.e., a mandatory yearly break from therapy with anti-CGRP pathway treatments (also referred to as drug holiday) to receive continued reimbursement for these therapies. Following Swiss reimbursement requirements, at the beginning of the study (during patient-recruitment phase), such treatment breaks had to be of a minimum duration of 3 months. However, during study conduct the mandatory therapy break was shortened to a minimum of 1 month, if patients showed a recurrence of migraine symptoms [3]. This article provides prospective data on the implementation and impact of this therapy interruption using MMD and mMIDAS results in a Swiss cohort of episodic and chronic migraine patients.

## Methods

### Study design

SQUARE is a 24-month, multicentric, non-interventional observational study conducted in Switzerland. After their written consent, eligible adult patients with episodic or chronic migraine were enrolled. The decision to treat with erenumab in accordance with the Swiss label [3] had been taken prior to study inclusion and was implemented independently of the study. The endpoints included:

- change from baseline in the number of MMD,
- change from baseline in the Headache Impact Test HIT-6™ score,
- change from baseline in the modified Migraine Disability Assessment (mMIDAS) with a 1-month recall period score (instead of 90 days to avoid overlapping MIDAS-scores due to time-flexibility of visits),
- change from baseline in the number of acute migraine-specific medication (AMSM) days, and
- change from baseline in the Impact of Migraine on Partners and Adolescent Children (IMPAC) score.

### Patients

To be enrolled in SQUARE, patients needed a diagnosis of migraine according to the International Classification of Headache Disorders (ICHD-3) [4], to sign an informed consent, and confirm to receive erenumab treatment in alignment with the Swiss label. Further, patients had to be willing to complete migraine diaries and patient reported outcome (PRO) questionnaires during the course of the study. Exclusion criteria comprised a prior treatment with erenumab or any GCRP (receptor)-based therapy, as well as any use of investigational drugs either during or within three months or five half-lives before study enrollment, which could alter the treatment effects of erenumab.

Of special note is that in the Swiss list of specialties (*Spezialitätenliste-SL*), erenumab has a regulated reimbursement [3]. In short, patients only qualify for reimbursed initiation of erenumab if they reach a minimum of 8 MMDs documented over at least 3 months and if they have had ≥ 2 prior prophylactic treatment failures in their medical history. For continued reimbursement, a reduction of MMDs must be observed under erenumab at month 3, reaching ≥ 50% reduction of MMDs at month 6.

In addition and as the focus of this work, a treatment interruption is mandated for all patients to continue reimbursement of erenumab beyond their first year, independently of the disease burden or erenumab response:

- Scheduling therapy interruption remains at the physician’s discretion, though it must occur at the latest 1 year after therapy start.
- If the patient suffers a recurrence of migraine burden during the interruption (≥ 8 MMDs in 30 days), cost-coverage of the resumption of the earlier anti-CGRP pathway therapy can be requested for another 12 months.
- Upon marketing authorization of erenumab in 2018, the therapy interruption had to have a minimum duration of 3 months. Notably, there was an amendment to this limitation in March 2021 (i.e., after the closure of the recruitment phase), which allowed to re-initiate erenumab as soon as the patient was suffering of more than 8 MMDs after treatment stop meaning a minimal duration of 1 month.

The majority of patients in this study interrupted therapy under the old reimbursement limitation, mandating a minimum interruption duration of 3 months [3].

In accordance with the Swiss list of specialties, the attending physicians interrupted treatment at their discretion depending on the treatment response and the improvement of the disease state. Thus, therapy interruption occurred at different time intervals and could have a different duration for the patients during the study period.

The impact of break and restart was investigated using data from PROs and patient diaries. In accordance with reimbursement regulations, control examinations are required at months 3, 6, and 12 after treatment initiation. The recommended visit schedule was chosen in accordance with these time points and reflects the timing after the day of the first injection of erenumab.

This interim analysis was conducted 18 months after the participants started erenumab treatment. Specifically, the impact of different lengths of drug-free periods on therapeutic outcomes was analyzed. For this observational study, descriptive methodologies were employed. Statistical tests confirmed the significance of all results with *p* values ≤ 0.05. No data imputations were conducted, accounting for the variations observed in the number of patients per visit and the calculated mean changes from baseline per endpoint, as illustrated in the figures. The terms therapy break and treatment interruption are used synonymously in this document.

## Results

A total of 172 patients (84.9% women) were enrolled from 19 sites across Switzerland between February 2019 and June 2020. Patients had a mean ± SD age of 44.2 ± 13.9 years, had experienced headaches for 28.2 ± 15.6 years and had been diagnosed with migraine for 18.6 ± 14.7 years. At baseline, patients had an average of 16.6 MMD (women 16.4; men 17.6). About 54% of the patients had EM, and the remaining 46% CM [2]. The majority of patients had experienced two or more prior preventive treatment failures (PPTF) [2], with propranolol/metoprolol, topiramate, antidepressants, and nutritional supplements mentioned as PPTF in ≥ 50% of patients, respectively [2, supplement].

**Fig. 1:**
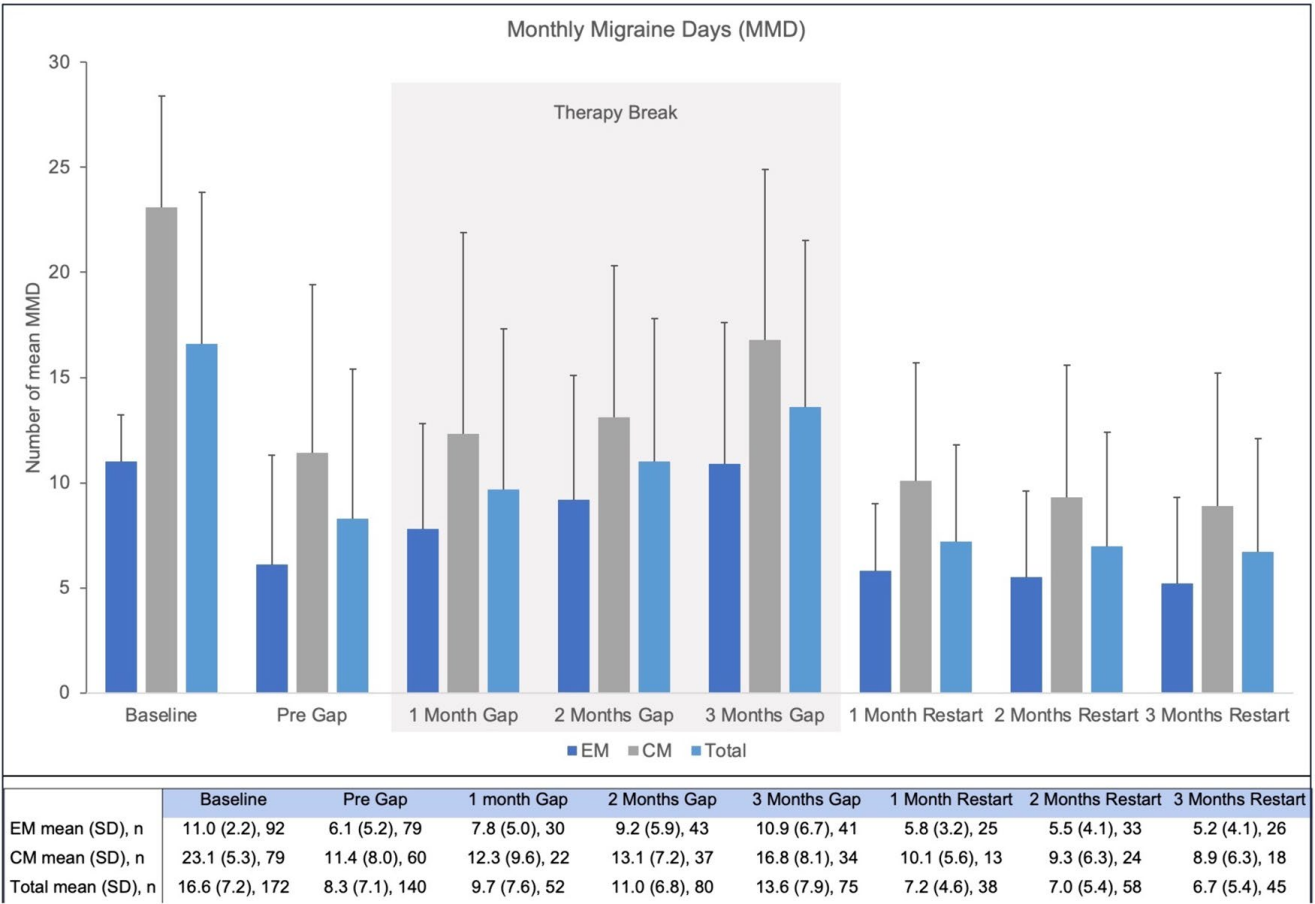
Impact of erenumab treatment on mean monthly migraine days (MMD) during the observational period of 18 months. *MMD* mean monthly migraine days; *Gap* therapy break; *EM* episodic migraine; *CM* chronic migraine; *Total* total study population; *n* number of patients; *SD* standard deviation

The baseline and 6-month interim results have previously been published [2]. The focus of this 18-month analysis is to investigate the impact of the treatment break. On average (± SD), the therapy break started 340.0 ± 49.4 days after treatment initiation and lasted for 115 ± 46.3 days. Briefly, patients experienced significant reductions in MMD from baseline to the start of therapy break, from 11.0 to 6.1 for EM and from 23.1 to 11.4 for CM (*p* < 0.001 for both groups, Fig. 1).

During the interruption period, migraine symptoms worsened: MMD increased toward baseline levels (10.9 and 16.8 at three months into therapy break for EM and CM, respectively), as shown in Fig. 1. The number of acute migraine-specific medication days (AMSM), which had dropped from initially 11.6 ± 7.0 at baseline to 6.6 ± 5.4 days at month 6 [2], also raised again to 9.8 ± 7 days during the treatment break at month 15 (data not shown).

**Fig. 2:**
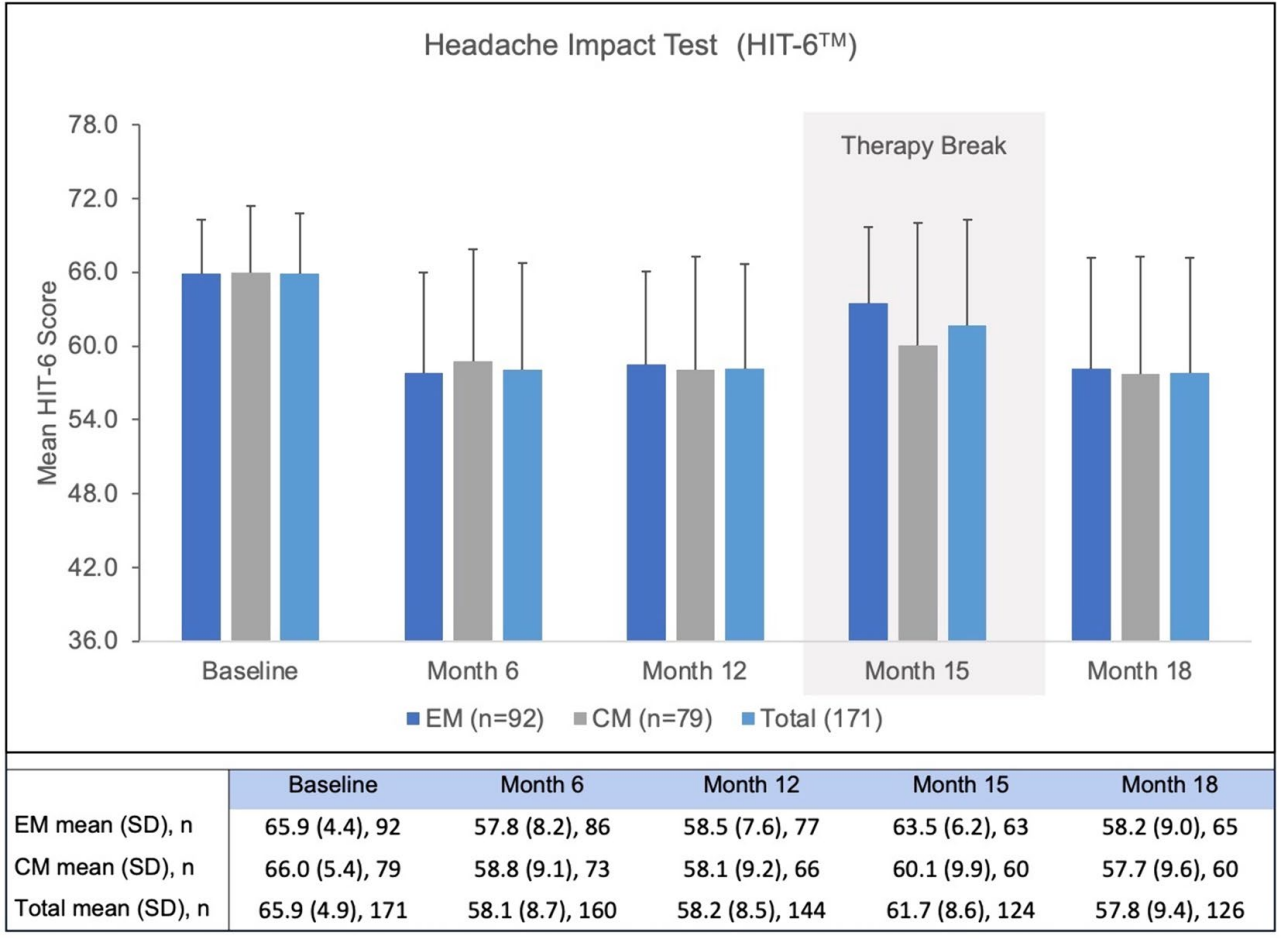
Impact of erenumab treatment on HIT-6™ during the observational period of 18 months. The full range of possible scores is shown, with 36 and 78 being the lowest- and highest-possible scores. *HIT* Headache Impact Test; *EM* episodic migraine; *CM* chronic migraine; *Total* total study population; *n* number of patients; *SD* standard deviation

The mean HIT-6™ score at baseline was 65.9 ± 4.9 and decreased significantly to 58.2 ± 8.5 (*p* < 0.001) over one year treatment period (Fig. 2). During treatment break, overall HIT-6^TM^ scores increased to 61.7 ± 8.6 (Fig. 2) and decreased again after resumption of erenumab therapy.

**Fig. 3:**
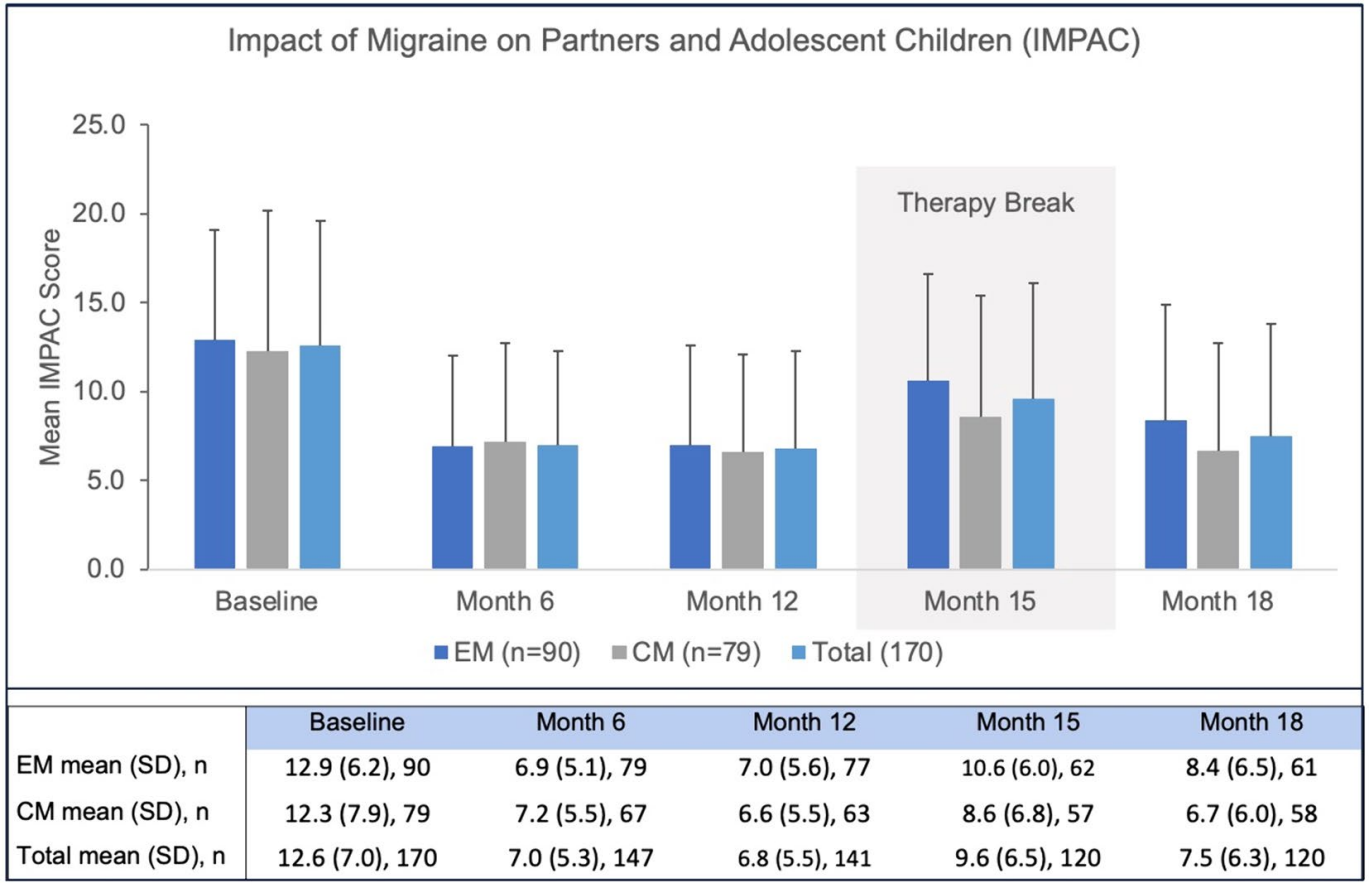
Impact of erenumab treatment on IMPAC during the observational period of 18 months. *IMPAC* Impact of Migraine on Partners and Adolescent Children; *EM* episodic migraine; *CM* chronic migraine; *Total* total study population; *n* number of patients; *SD* standard deviation

Overall IMPAC score decreased significantly from 12.6 ± 7.0 to 6.8 ± 5.5 (*p* < 0.001) by month 12. Treatment interruption by month 15 was characterized by an increase in IMPAC score to 9.6 ± 6.5, which decreased to near pre-break values of 7.5 ± 6.3 (*p* < 0.001) by the next visit at month 18 after treatment reuptake (Fig. 3).

**Fig. 4:**
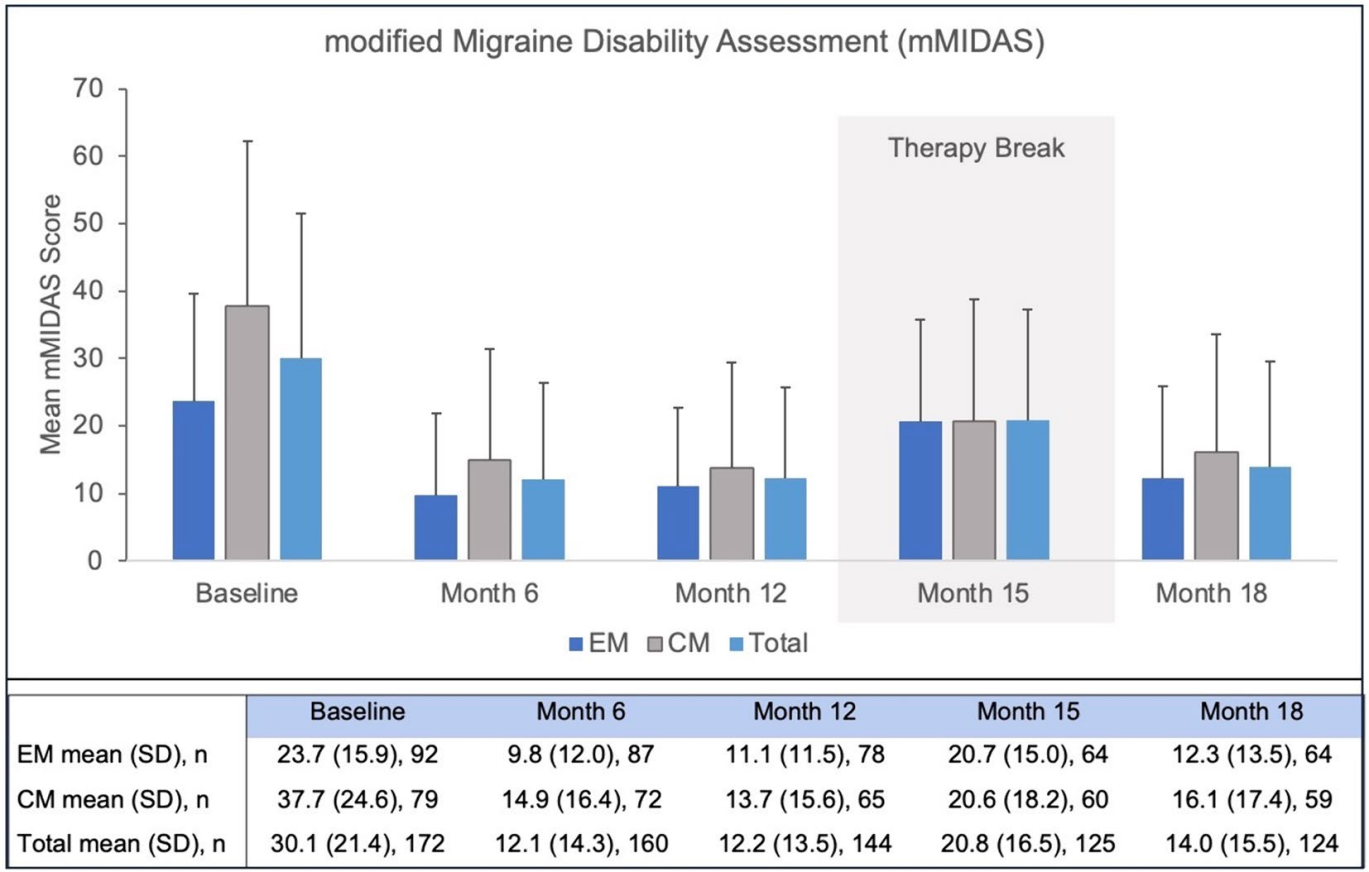
Impact of erenumab treatment on mMIDAS during the observational period of 18 months. *mMIDAS* modified Migraine Disability Assessment; *EM* episodic migraine; *CM* chronic migraine; *Total* total study population; *n* number of patients; *SD* standard deviation

Similarly to previously mentioned endpoints, the impact of the therapy break was also evident in the mMIDAS scores: baseline scores of 30.1 ± 21.4 decreased to 12.2 ± 13.5 at month 12 and increased again to 20.8 ± 16.5 at month 15 when patients went into treatment break (Fig. 4). After therapy resumption, patients were able to re-achieve pre-interruption results (Figs. 1, 2, 3, 4).

**Figure 5:**
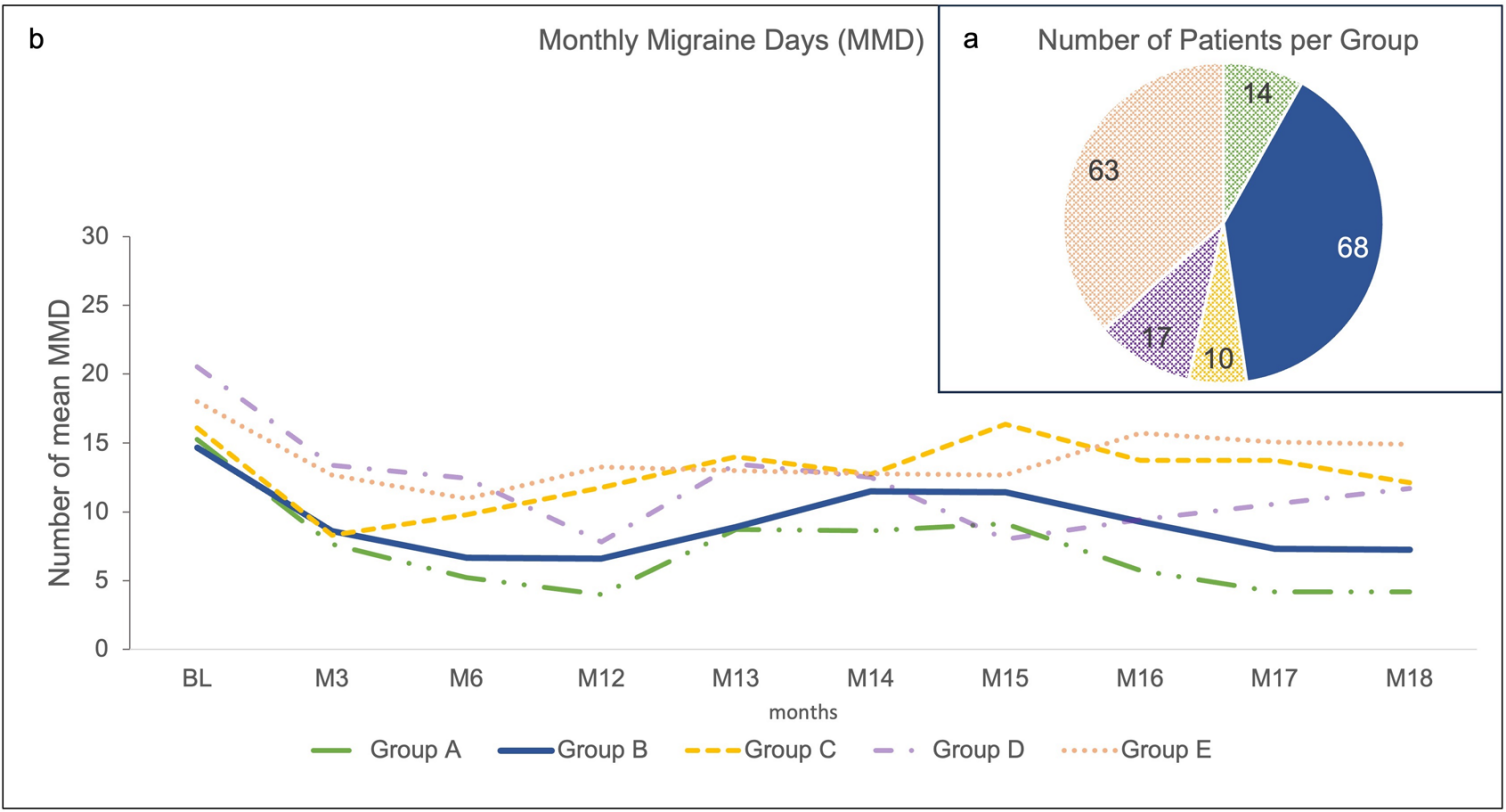
**a** Number of patients per group; **b** Impact of erenumab treatment break on MMD per group over 18 months. Group A (*n* = 14): > 0 to < 2.5 months break; Group B (*n* = 68): ≥ 2.5 to < 6 months break; Group C (*n* = 10): ≥ 6 months break (with a therapy restart); Group D (*n* = 17): no break; Group E (*n* = 63): no documented therapy re-initiation during observational period (therapy break/no reuptake data available). *BL* baseline; *M* month; *MMD*, mean monthly migraine days.

As the length of the treatment break was differing among patients, they were grouped with regards to therapy break duration. Patients fell into the following groups (Fig. 5a and b): Group A had a break duration of up to 2.5 months (*n* = 14), group B between 2.5 and < 6 months (*n* = 68). Group C had a therapy break of ≥ 6 months and restarted therapy (*n* = 10), group D had no break (due to individual reimbursement settings, *n* = 17), and group E with no documented therapy re-initiation during the observational period (therapy break/no restart data available, *n* = 68). The majority of patients fell into group B and group E, see Fig. 5a.

Group B had a baseline MMD score of 14.6, which decreased to 6.7 at month 3 of the observational period. During treatment break, which majorly comprised months 13 to 15 of the study period, MMD scores increased to 8.9 in month 13 and up to 11.4 in month 15. Upon treatment resumption, MMD scores decreased again to 9.2 in month 16 and further to 7.2 in month 18 (Fig. 5b). Groups A and B reached visible treatment benefits from baseline to month 12 with regards to reduction of MMD (from 15.2 to 4.0 MMD for Group A and from 14.6 to 6.6 MMD for Group B, Fig. 5b). Further, these groups experienced similar effectiveness of erenumab after treatment resumption, with MMD numbers settling at those before treatment interruption (4.1 and 7.2 respectively for group A and B at month 18, Fig. 5b). This pattern was not observed in patients with ≥ 6 months off therapy (group C). Groups D and E had higher MMD levels at baseline and similarly to group C also did not show sustained MMD reductions over the observational period.

**Fig. 6:**
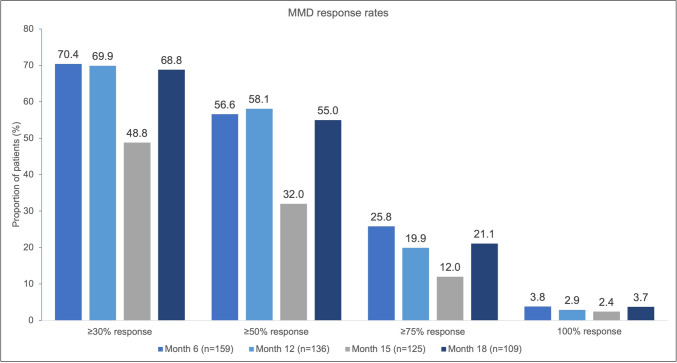
Effect of erenumab treatment on mean monthly migraine days (MMD) response rates. *MMD* mean monthly migraine days; *n*, number of patients

The impact of therapy break is also evident when grouping the study population by the percent reduction of MMD: Fig. 6 describes the number of patients who achieved a ≥ 30%, ≥ 50%, ≥ 75% and 100% reduction in MMD from baseline at 6-, 12-, 15-, and 18-month visits.

Overall, during erenumab treatment, response rates to therapy were similar at all appointed study visits at month 6, 12, and 18: approximately 70%, 57%, 22%, and 3.5% of the patients reached ≥ 30%, ≥ 50%, ≥ 75% and 100% MMD reduction respectively. The response rate of patients was lowest at the end of the obligatory therapy break, i.e., at month 15: 48.8%, 32.0%, 12.0% and 2.4% of patients achieved a ≥ 30%, ≥ 50%, ≥ 75% and 100% reduction in MMD, respectively.

## Discussion

This interim analysis investigates the impact of the mandatory interruption of erenumab therapy in adult patients with episodic and chronic migraine. The therapy was interrupted on average 11.2 months after initiation with a mean duration of four months; the effect of the break was most clearly visible at the month 15 visit (3 months gap). Overall, during the therapy break, the majority of patients experienced an increase in migraine frequency, necessitating the resumption of erenumab treatment. These findings are consistent with two previous retrospective real-world studies [5, 6]. Other published data also indicate that the frequency of migraine symptoms rises in patients upon treatment interruption [7]. This suggests that the effects of anti-CGRP antibody treatments such as erenumab do not have an impact beyond their treatment period.

Upon restart of therapy, mean monthly migraine days reached similar levels compared to the last visit before interruption. Similar observations were reported for the other parameters in the result section, HIT-6™, IMPAC, the mMIDAS score and associated quality of life. These results are in line with previous, which showed response to erenumab therapy after break [6]. Due to the reimbursement requirements, the SQUARE results do not allow to compare continuous treatment with interrupted treatment as it was investigated in the previous study. However, they confirm a response with treatment restart after the break. The initial effectiveness of erenumab therapy recovered by month 18 following interruption, especially in those patients with < 6 months break duration. Further, the proportion of patients achieving ≥ 30%,  ≥ 50%,  ≥ 75%, and 100% response in MMD reductions after therapy resumption at month 18 is comparable to the proportions achieved prior to treatment break.

Considering the profound impact of recurrent migraine attacks on patients with migraine diagnosis [8], it becomes a medical, social, and economic necessity to prioritize the prevention of a resurgence in migraine frequency. Consequently, the duration of the interruption should be minimized to the greatest extent possible to allow the physician a broader possibility for individual patients’ assessment.

Out of the total study population, only a small group of 14 patients (group A) took a break of less than 3 months, which might be related to the change in reimbursement criteria. Due to the small number of patients, the probability of a meaningful influence on the overall study results is low, and pointing out a favorable treatment-break duration is difficult.

However, it is evident from our data that it takes less than three months to observe a worsening of disease, as shown as rise in MMD and mMIDAS. Patients who experience a rapid symptom worsening when going into the treatment break might fulfill the criteria for reimbursement of therapy restart already after 1 month of break. The reimbursement amendment could therefore be favorable concerning the physician’s scope for action.

Patient numbers in groups C and D are very low, making these data difficult to interpret: This group individuality is reflected in the wide range and different lengths of therapy gaps in the study population, revealing the heterogeneity of response to the break in a real-life setting. Final study results after 24 months might provide more details on these groups and especially the group of patients with no documented therapy restart within the 18 months observational timeframe (group E).

The strongest argument in favor of a treatment gap is the possibility of evaluating the continuous need for long-term treatment, such as erenumab, targeting the CGRP pathway. As a therapy interruption puts considerable burden on many patients, the exact timing and duration must be carefully weighed on an individual basis.

The results after study end (24 months) will reveal overall therapy performance in patients with episodic and chronic migraine and the influence of the therapy break on treatment efficacy and therapy success over time. Also, the effects on healthcare costs (e.g., emergency visits or acute medication use) will be further analyzed.

## Abbreviations

AE: Adverse event
CGRP Receptor: Calcitonin gene-related peptide Receptor
CM: Chronic migraine
eCRF: Electronic Case Report Form
EKNZ: Ethikkommission Nordwest- und Zentralschweiz
EM: Episodic migraine
HIT-6™: Headache Impact Test
IMPAC: Impact of Migraine on Partners and Adolescent Children
MMD: Monthly migraine days
mMIDAS: Modified Migraine Disability Assessment
PRO: Patient-reported outcome
RAPS: Registry of All Projects in Switzerland
SD: Standard Deviation
SQUARE: Swiss QUality of life and healthcare impact Assessment in a Real-world Erenumab treated migraine population

## Declarations

### Availability of data and materials

In adherence to the principles of open science and transparency, the data supporting the findings of this study are available to researchers on reasonable request for purposes of replication or data re-use.
